# Injectable hyaluronic acid-based hydrogel niches to create localized and time-controlled therapy delivery

**DOI:** 10.1016/j.mtbio.2025.101510

**Published:** 2025-01-24

**Authors:** Veronica Torresan, Alessandro Gandin, Paolo Contessotto, Francesca Zanconato, Giovanna Brusatin

**Affiliations:** a Department of Industrial Engineering, University of Padova and INSTM, Via Marzolo 9, Padova, 35131, Italy; b Department of Molecular Medicine, University of Padova, Via Ugo Bassi 58/B, Padova, 35131, Italy

**Keywords:** Injectable hydrogels, Hydrogel niches, Therapies delivery

## Abstract

The use of hydrogel-based niches for therapy delivery enables the concentration of active components and cells in a targeted area. This approach enhances efficacy while minimizing systemic side effects by spatially controlling the release of the therapy. Precise tuning of the matrix's chemical properties and control of both material degradation and release profile of biologically active components are required to reduce the optimal dose and extend its therapeutic effect. Here we aimed to develop an injectable hydrogel that can fulfill all these requirements. We designed a system based on hyaluronic acid, crosslinked via click-reaction with multi-arm polyethyleneglycol and functionalized with RGD peptides. Additionally, we incorporated thiol-modified heparin into the formulation, which provides specific binding sites for cytokines. Our results indicate that heparin incorporation can delay cytokine release, while the release of nanocarriers can be regulated by adjusting the crosslinking degree. This design modulates pore size and degradation time, while preserving the injectability of the niche. In conclusion, this system offers a versatile and efficient delivery platform suitable for therapeutic applications in a wide range of diseases.

## Graphical abstract

**Figure:**
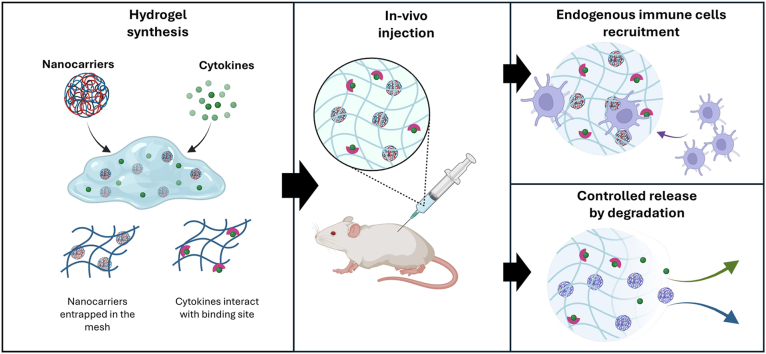
This images was created using Biorender.

## 1 Introduction

Localized therapies delivery is based on the administration of drugs directly to a specific site within the body, rather than releasing them systemically throughout the patient's body. This approach aims to achieve a prolonged and controlled release of the therapeutic, by reducing treatments doses, and thus minimizing systemic side effects and toxicity [1].

One of the most promising approaches in this area is immunotherapy, especially therapeutic vaccines [2]. These vaccines are designed to precisely instruct the patient's own innate and adaptive immune system to either prevent or treat diseases. Vaccine immunotherapies offer significant potential for addressing a wide range of conditions, including cancer, chronic infections, and autoimmune disorders [3]. The effectiveness of these therapies often depends on the delivery of various immunomodulatory molecules and adjuvants, such as cytokines, nucleic acids, small molecules, antigens, and peptides. To protect sensitive biomolecules from degradation such as RNA, their delivery is typically accomplished using specialized nanocarriers. Additionally, current vaccine immunotherapies frequently involve the recruiting of immune cells (e.g., dendritic cells [1,4]) to the delivery site, to efficiently mount an immune response.

In addition to vaccine immunotherapy applications, cell-based treatments, which use patient-derived immune cells, such as T-cells, NK or dendritic cells, can be used as therapeutic agents incorporated into the vaccine formulation.

In all these scenarios, a biomaterial needs to be engineered to create a niche for the local recruitment or delivery of cells along with the therapy formulation [3].

This biomaterial niche would spatially confine and protect active components, and control their release from the injection site [3], leading to a more effective and less toxic treatment at lower doses. Additionally, it helps in supporting the viability of any embedded or recruited cells. Furthermore, the versatile nature of the niche allows for the combination of therapies to provide a synergistic effect, while its localized administration around the affected area, such as a tumor, can be strategically utilized to maximize the therapeutic impact.

For developing the injectable niche, hydrogels are among the most promising biomaterials [[5], [6], [7]]. They better mimic the cellular microenvironment compared to other biomaterials and can be engineered to be injectable and sufficiently soft, thereby minimizing foreign body response and reducing the need for surgical intervention [8].

Significant progress has been made in this field, particularly by Mooney and colleagues, who have demonstrated the potential of hydrogel-based systems in cancer immunotherapy [[9], [10], [11], [12], [13], [14]]. Their research focused on the design of biomimetic hydrogel scaffolds that can modulate immune responses and guide tissue regeneration. Among their optimized systems, cryogels function as an injectable sponge for the delivery of cells and adjuvants. These systems offer numerous advantages, but they lack versatile strategies for delivering different active components, such as viable cells (e.g. dendritic cell-based therapy), small molecules, and chemokines or cytokines. For instance, while the injectable-cryogel have demonstrated significant results in eliciting immune response, the cytokines release reaches the 80 % after only 2 days [15]. To address this issue, cytokines were complexed with PEI, in order to form cationic nanoparticles that can interact with the cryogel. However, the release requires the use of ultrasounds due to the lack of alginate in-vivo degradation [11]. Additionally, chemicals used in these systems are not FDA-approved, leading to possible issues in the in-vivo application and the absence of adhesive motifs for endogenous and exogenous cells makes these systems unsuitable for cell-based therapies [11,12,15]. Despite its efficacy, this vaccination system faces other limitations, such as slow in-vivo degradation and high stiffness, in the order of thousands of Pa, which can trigger foreign body reaction [16]. To address these limitations, different strategies have been proposed [8]. Burdick et al. [17] developed an injectable shear-thinning hydrogel based on hyaluronic acid (HA) using guest-host chemistry. While this system improves injectability and degradability, its synthesis remains complex, requiring long purification steps to eliminate residual reaction molecules. Additionally, the study primarily focus on protein release, without investigating alternative strategies for the release of small molecules or chemokines/cytokines [17].

Here, we developed a soft hydrogel scaffold based on hyaluronic acid (HA) functionalized with RGD peptides and incorporating heparin [18]. This hydrogel is mildly crosslinked with polyethylene glycol (PEG) using a biocompatible click reaction between thiols and acrylate groups. The novelty of our formulation, developed by adapting a previously reported crosslinking strategy [19,20], lies in its broader combination of advantageous characteristics compared to existing alternatives. These include the injectability, softness (to minimize foreign body reactions), tunable drug release (enabled by adjustable porosity and the incorporation of heparin), and the use, exclusively, of FDA-approved components. These features collectively ensure its suitability for potential in-vivo applications and scalability. This optimized design results in a highly customizable platform capable of the simultaneous delivery of: viable cells (through RGD functionalization), chemokines or cytokines (via heparin incorporation) and small molecules encapsulated in nanocarriers (due to its tunable nanometer-scale mesh size). Additionally, the hydrogel supports controlled release through compatible in-vivo degradation. As a proof of concept, we evaluated this HA-based hydrogel in both in-vitro and in-vivo settings, demonstrating its potential as an effective tool for creating localized biomaterial niches suitable for a wide range of targeted therapy delivery applications.

## 2 Results and discussion

### 2.1 Design and characterization of injectable hydrogel

To design our immunomodulatory niche for the controlled loading and release of therapeutic components, such as molecules, nanocarriers and cells, we engineered a biomaterial that fulfills several key criteria. Firstly, it needs to have sufficient rigidity to establish a local niche capable of withstanding tissue pressure, while remaining soft enough to avoid recognition as a foreign body and, if needed, to safely encapsulate cells. Secondly, it should exhibit appropriate porosity and cell adhesiveness to facilitate immune cell infiltration in-situ or enable cell delivery. Finally, it must offer precise spatiotemporal control over the release profiles of drugs and nanocarriers.

This last requirement can be addressed through different strategies, including controlling the gel's degradation to regulate the release of physically entrapped molecules and nanocarriers; leveraging chemical affinity between the gel and small molecules to modulate their release; employing covalent coupling of active components to ensure their long-term availability until the scaffold is fully degraded [1].

Another key consideration is the delivery method of the biomaterial niche, which can be either surgically implanted or locally injected. The latter is preferred for its minimally invasive nature, but it requires the development of an injectable gel. Various approaches can be employed, such as gels that form in-situ through thermogelling or chemical crosslinking, as well as shear-thinning materials [21,22]. Although shear-thinning and thermogelling hydrogels limit the range of suitable materials, in-situ crosslinking hydrogels that utilize biocompatible reactions offer greater versatility in terms of composition.

#### 2.1.1 Hydrogel niche synthesis

Based on these considerations, we developed an in-situ forming hydrogel scaffold using crosslinked hyaluronic-acid (HA), a biocompatible and in-vivo degradable gel that can be chemically modified to introduce functionalities (e.g. cell adhesive groups) or to modulate crosslinking degree to tune rigidity and injectability.

**Fig. 1:**
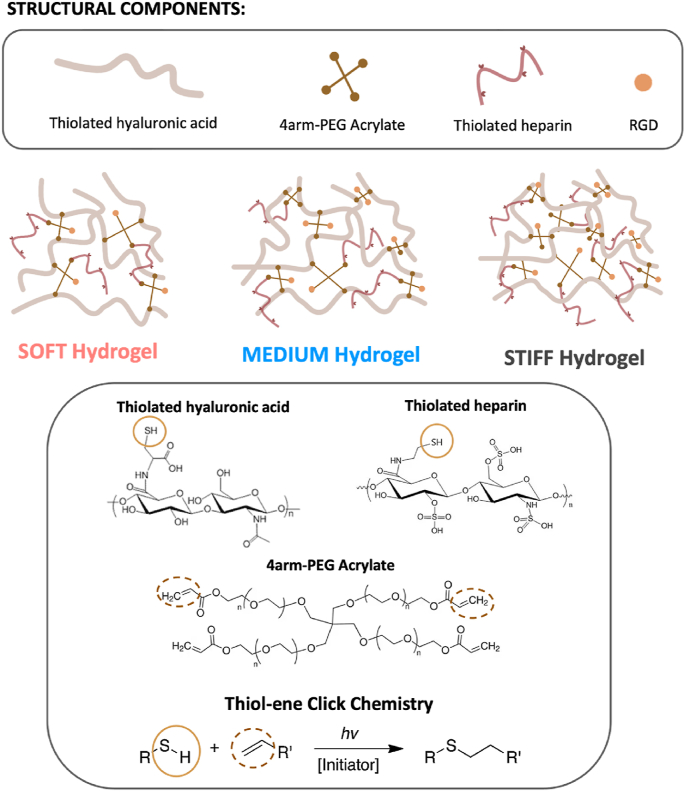
Representation of the hydrogel components, their chemical structure and their 3D interconnection in the soft, medium, and stiff compositions. The figure also includes the reaction scheme of the thiol-ene click chemistry selected.

In this study, we utilized a thiol-modified hyaluronic acid crosslinked with a 4-arm acrylate polyethyleneglycol (PEG-4-arm-Ac) to create a gel through a biocompatible thiol-ene click reaction between thiols and acrylate groups. Fig. 1 presents a schematic representation of our injectable material, with the specific hydrogel compositions detailed in Table S1.

By optimizing the crosslinking dynamics and hyaluronic acid concentration, this gel can be prepared as an injectable formulation that remains localized at the injection site, forming a depot for the sustained and controlled release of therapy components over extended and adjustable timeframes. As we will demonstrate, this approach enables precise control over the release of encapsulated components via diffusion or degradation mechanisms.

In addition, we improved our scaffold by incorporating thiol-modified heparin (Hep), which provides binding sites for a range of cytokines and chemokines used in different therapies [[23], [24], [25], [26], [27]].

To support the embedding of exogenous cells and promote the infiltration of endogenous immune cells, we further functionalized the material with a synthetic cell-adhesive peptide. Specifically, PEG-4-arm-Ac was pre-functionalized with a RGD peptide, a synthetic adhesive sequence derived from fibronectin, and then it was used as a crosslinker for the thiolated hyaluronic acid. In our synthesis, a mean of 2.5 out of 4-arm-Ac were occupied by the peptide, maximizing RGD functionalization of the gel while leaving enough arms free for subsequent crosslinking with thiolated-HA. Therefore, our study focused on three different formulations, each characterized by varying degrees of crosslinking, a key parameter that primarily influences these properties. These injectable hydrogels were classified as soft, medium, and stiff based on their stiffness, which is directly related to the crosslinking degree. To achieve the desired stiffness while maintaining injectability, we increased the PEG-4-arm-Ac content relative to HA and made slight adjustments to the hyaluronic acid concentration.

To confirm the successful reaction between the acrylates and the thiols groups, we performed the Ellman's test, a standard assay for the determinations of free thiols. Specifically, we tested the RGD solution before its reaction with the PEG, and the final solution resulting from the mix of the two components (referred as premix). As shown in Fig. S1, the peak corresponding to the revealed free -SH groups was observed only in the unreacted RGD solution. Conversely, no peak was detected in the premix solution, indicating that all the RGD peptide was successfully conjugated to the PEG (Fig. S1).

#### 2.1.2 Rheological behaviour

**Fig. 2:**
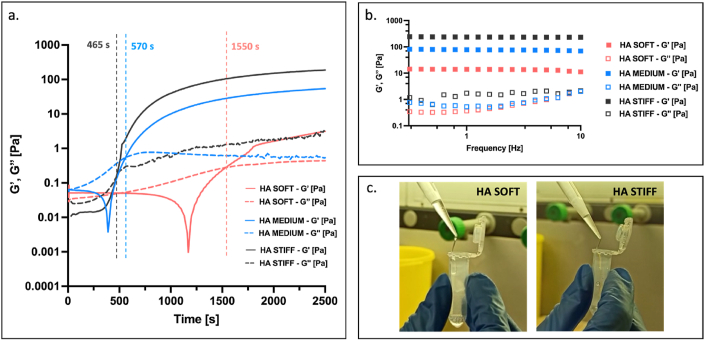
**a.** Dynamic time sweep of soft (pink), medium (blue) and stiff (black) HA hydrogels. In the graph, G’ (storage modulus) is represented with a solid line while G” (loss modulus) is represented with a dashed line and their crossover determines the gelling point at 465 s for HA stiff, 570 s for HA medium and 1550 s for HA soft. The preceding decrease of the storage modulus in the soft and medium stiffness compositions is an instrumental effect due to rheometer head inertia dominating the sample feedback while still a low viscosity liquid; **b.** Frequency sweep of the investigated formulations: soft hydrogel (pink squares), medium hydrogel (blue squares) and stiff hydrogel (dark squares). G′ is represented with solid squares while G″ with empty squares; **c.** Injectability of the designed HA hydrogel. Images were taken at the gelling point defined by the crossover in Fig. 2a for the HA soft (on the left) and HA stiff (on the right). (For interpretation of the references to colour in this figure legend, the reader is referred to the Web version of this article.)

The rheological properties of the hydrogels are critical to control gelling time and injectability. To monitor the temporal evolution of the storage modulus (G′) and loss modulus (G″) we performed dynamic time sweep test over a period of 1 h. This approach enabled us to determine the gelling time for each formulation and identify the optimal timeframe in which the hydrogel maintains its injectable behavior. Our results reveal that the gelling point, defined by the crossover of G′ and G″, varies from 1′550 s (approximately 26 minutes) for soft gels to 465 s (approximately 8 minutes) for stiff gels, as shown in Fig. 2a. The gels were injected a few minutes after reaching the gelling point, at which the gel starts to slightly crosslink. This timeframe is essential to stabilize the gel prior to injection, ensuring its confinement and the formation of a localized depot for therapeutic components [3,14].

Next, we measured the final moduli using frequency-sweep test within the linear viscoelastic regime (LVR) at a frequency of 1 Hz. The storage moduli (G’) for soft, medium and stiff gels were determined to be of 14 Pa, 78 Pa and 238 Pa, respectively, as shown in Fig. 2b. To confirm the injectability of each formulation, we loaded the gels into a micropipette tip and extruded them within the timeframe established by the dynamic time sweep measurements. In Fig. 2c is reported, as an example, the extrusion of soft and stiff hydrogel, and in Movie S1 (Supporting Information) the video of the injectability of stiff hydrogel, recorded at the gelling point. These findings demonstrated that gelling time and stiffness can be precisely adjusted by modifying the crosslinker degree and hyaluronic acid concentration. This enables the creation of injectable gels with customizable physical properties.

#### 2.1.3 Mesh size

The hydrogel microstructure plays a critical role in cell and nanocarrier encapsulation, as well as in controlling their release through hydrogel degradation or drug diffusion. In particular, the mesh size has a significant impact on the entrapment of nanocarriers and the diffusion of small molecules. To assess the average mesh size, we applied the principles of rubber elasticity theory, which describes the mechanical behavior of elastomeric materials. According to this theory, the storage modulus (G′), indicative of the material's intrinsic elasticity, is inversely related to the cube of the mesh size. This correlation allows the calculation of the mesh size from experimental G′ measurements [28]. Our findings indicate that, under the synthesis conditions described in Materials and Methods, the mesh size can be adjusted from 67 nm, for soft hydrogel, to 37 nm, for medium hydrogel, and to 26 nm for stiff hydrogel, as summarized in Table 1.

**Table 1:** In the table are reported the G’ values at 1 Hz of frequency and the relative mesh size values obtained with Equation (1) (see Experimental section).

HYDROGEL	G’ [Pa] – 1Hz	Mesh Size
**SOFT**	14	67 nm
**MEDIUM**	78	37 nm
**STIFF**	238	26 nm

As expected, we found a decrease in mesh size with increasing crosslinker degree and polymer concentration. To prevent the burst release of the nanocarriers and ensure their physical entrapment, we used nanocarriers with a diameter larger than 67 nm. This approach ensures that their release is governed solely by the degradation of the surrounding hydrogel niche. The porous structure of the hydrogel was confirmed through SEM analysis, as shown in Fig. S2. The differences in pore sizes observed in the SEM images compared to those calculated using rubber elasticity theory can be attributed to the processing steps involved prior to SEM analysis. Specifically, the freezing method employed may have affected the hydrogel's porosity, as reported in literature [29].

#### 2.1.4 Nanoparticles and cytokines release

To function effectively as a localized biomaterial niche, our gel must efficiently encapsulate and release therapeutics components and nanocarriers in a controlled manner. These nanocarriers can be loaded with biomolecules, cytokines, adjuvants or nucleic acids [30], protecting them from enzymatic degradation in-vivo, while enhancing their solubility, bioavailability, and cellular uptake, thereby enabling targeted delivery to specific cells [31,32]. Additionally, co-delivering biomolecules within nanocarriers allows for greater control over their release, primarily influenced by charge or steric interactions that immobilize the nanocarriers within the niche. As a consequence, by using nanocarriers we can significantly improve therapy efficacy by protecting adjuvants from degradation and modulating the drug release profile, thus preventing the initial burst and prolonging the therapeutic effect [33].

**Fig. 3:**
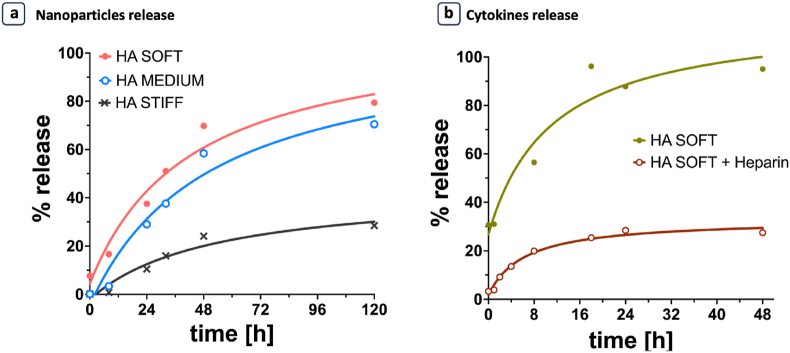
**a.** Percentage of release of fluorescent 100 nm diameter polystyrene nanoparticles from a soft (full dots), medium (empty dots) and stiff hydrogel (cross); **b.** Percentage of release of IFN-γ from soft hydrogel with heparin binding sites (empty dots) and without (full dots). Each condition was done in triplicate, and the values of mean and standard deviation are reported in Tables S2 and S3.

We therefore investigated the impact of mesh size on nanoparticles release and the effect of heparin functionalization on the cytokines release. To examine the release behavior of nanocarriers in soft, medium, and stiff hydrogel formulations, we used polystyrene (PS) nanoparticles (NPs) with a diameter of 100 nm, which is larger than the pore sizes in our gels (ranging from 26 to 67 nm). FITC-PS nanoparticles (100 nm) were embedded in the hydrogels, and the fluorescence intensity of the supernatant was analyzed at different timepoints (0, 8, 24, 32, 48 and 120 hours). The release of NPs (Fig. 3a) followed the trend of polymer mass loss, and no burst effect was observed in the initial hours. Specifically, soft and medium hydrogels exhibited a similar kinetic profile, while stiff hydrogels never achieved a complete release, reaching around 30 % release after 120 h, compared to over 60 % for the other two formulations. It is important to note that in this study, NP release was determined solely by the degradation rate. Indeed, there were no chemical or physical interactions between the NPs and the gel, and the mesh size of the gels is smaller than NPs size preventing their rapid diffusion out of the matrix. Moreover, given the frequent use of cytokines in therapeutic treatment, we assessed also the release of these biomolecules. As previously mentioned, heparin naturally presents binding sites for many cytokines [[23], [24], [25], [26], [27]] We hypothesized that incorporating heparin in our formulation would enhance cytokine retention within the niche. To test this hypothesis, we incorporated IFN-γ into a heparin-functionalized soft HA hydrogel and compared it to a soft HA hydrogel without heparin, which had the largest pores size. We monitored the concentration of IFN-γ released in the solution over 2 days at different timepoints (0, 1, 8, 18, 24 and 48 hours) using an ELISA assay. The results showed that heparin functionalization effectively controls cytokines release, significantly slowing the release of IFN-γ compared to the control without heparin. After 48 h, only the 30 % of IFN-γ was released in the heparin functionalized hydrogel, compared to 100 % in the control (Fig. 3b). This approach allows for extended availability of immunomodulatory cytokines with high affinity for heparin binding sites (such as IFN-γ, GM-CSF, CXCL-10, and others) to immune cells.

#### 2.1.5 Cell embedding and viability

To demonstrate that our gels can support 3D cell viability, we embedded immortalized human mammary epithelial cells (MCF10A) within soft and stiff gels containing adhesive motif (RGD peptide) and monitored their morphology over 14 days. In both cases, we observed the formation of spheroid structures (Fig. S3), which continued to grow in size until the final time point (day 14). These results indicate that cell proliferation, and consequently cell viability, is comparable regardless of the gel formulation and stiffness.

**Fig. 4:**
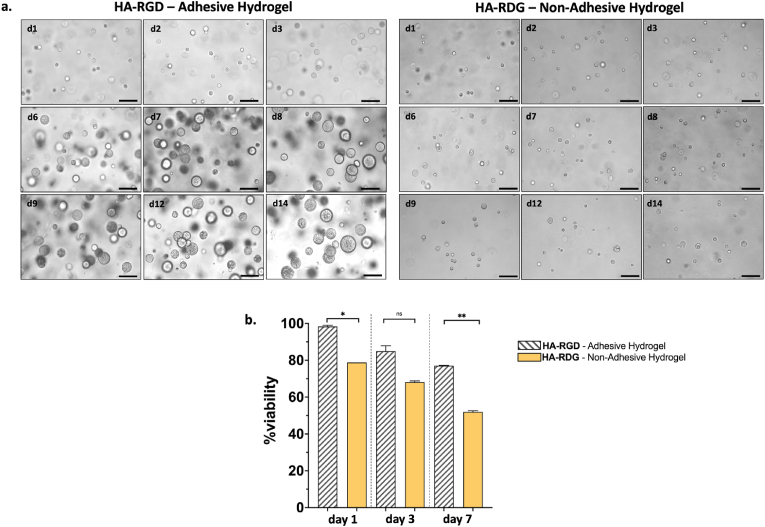
**3D cell culture in HA stiff hydrogel and viability assay**. **a.** MCF10A single cells embedded in HA-RGD Adhesive Hydrogel and HA-RDG Non-Adhesive Hydrogel for 14 days. In the HA-RGD hydrogel, formation of spheroids is detected from day 3 while in HA-RDG hydrogel spheroids formation is never observed. Regarding cell viability, higher values are always observed in the cells embedded in the adhesive hydrogel. Each condition was made in duplicate. Scale bar = 100 μm. **b.** Viability assay on MCF10A cultured in HA-RGD and HA-RDG hydrogels. Statistically significant differences were evaluated comparing two groups by Welch's *t*-test and considering a confidence interval of 95 % (*p* = 0.0147 in column 1 vs 2; *p* = 0.0661 in column 3 vs 4; *p* = 0.0069 in column 5 vs 6). Data show the mean and SD.

Next, to assess the specific role of the RGD peptide, we embedded the same cell line in a stiff gel containing either the adhesive motif (RGD peptide) or a non-adhesive variant (RDG peptide) and we monitored their morphology over 14 days. As shown in Fig. 4a, cells embedded in HA-RGD matrix formed spheroids from day 3. Additionally, these spheroids continued to grow in size until day 14, confirming their viability until the endpoint. In contrast, cells embedded in the HA-RDG matrix displayed a single-cell morphology with reduced viability observed from day 1. Live and dead staining data (Fig. 4b) indicated high cell viability up to day 7 in HA-RGD hydrogel. However, as the spheroids grew in diameter over time, this limited our ability to accurately quantify viable single cells given the high-density. This confirms that RGD functionalization is essential for MCF10A attachment and proliferation within the HA hydrogel.

**Fig. 5:**
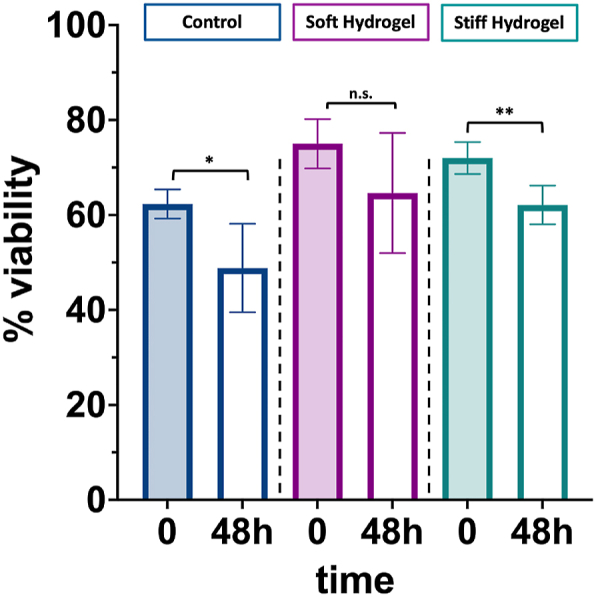
Cell viability of splenocytes cultured in suspension (Control, blue) or embedded in a soft or stiff hydrogel (Soft hydrogel, magenta; Stiff hydrogel, green). Each sample was made in duplicate. Statistically significant differences were evaluated comparing two groups by Welch's *t*-test and considering a confidence interval of 95 % (*p* = 0.0159 in column 1 vs 2; *p* = 0.0614 in column 3 vs 4; *p* = 0.0026 in column 5 vs 6. Data show the mean and SD. (For interpretation of the references to colour in this figure legend, the reader is referred to the Web version of this article.)

We also studied the viability of primary cells embedded in the soft and stiff hydrogel for 48 h. Specifically, we investigated the viability of splenocytes to evaluate the ability of hydrogels to host primary immune cells. Indeed, our formulations were designed to support both the delivery of exogenous immune cells and the infiltration of endogenous immune cells. Our findings indicated that the viability of mouse splenocytes within both soft and stiff hydrogels was comparable to that of cells in standard suspension culture, with both conditions exhibiting a minimal 10 % reduction in viability after 48 h (Fig. 5). This result further demonstrates the cytocompatibility of the developed injectable hydrogel, regardless of its stiffness.

In summary, the chemical design of this HA hydrogel enables precise tuning of the crosslinking degree and rheological properties, which in turn controls the degradation time while maintaining injectability. We demonstrated that the release of NPs can be regulated by modifying the HA crosslinking degree or concentration, and that cytokines release can be effectively controlled through heparin functionalization. Moreover, RGD functionalization facilitates cell embedding and adhesion, preserving cell viability regardless of the hydrogel's stiffness.

### 2.2 *In-vivo* gel behaviour

A typical whole therapeutic formulation might include: (i) cells (ii) cytokines and chemokines to enhance specific cell signaling and (iii) nanocarriers containing adjuvants and small molecules. Our hydrogel was specifically designed and synthesized to accommodate all these components. This was achieved through the incorporation of RGD motifs for cell adhesion, heparin for cytokines binding, and tunable mesh size for nanocarriers encapsulation. Additionally, the stiffness and degradation rate of the hydrogel were optimized to control the release of all therapeutic components. All these characteristics are compatible with in-vivo applications due to the use of biocompatible precursors and the click crosslinking reaction.

As a proof of concept to validate the in-vivo performance of our hydrogels, we first injected various hydrogel formulations and evaluated the tissue response to localize injection and degradability.

#### 2.2.1 In-vivo tissue response to the hydrogel

We first compared the in-vivo behaviour of our HA hydrogel niche with a porous hydrogel widely used in biomaterial-based vaccines by Mooney et al. [10,15], named cryogel. Their studies demonstrated that a combination of PEG and alginate can be engineered into a microporous biomaterial capable of recruiting and instructing immune cells, effectively inducing robust antitumor immune responses. In our study, equal volumes of either cryogel or HA stiff hydrogel were injected intraperitoneally (IP) into mice, and the remaining mass was recovered after 72 h.

**Fig. 6:**
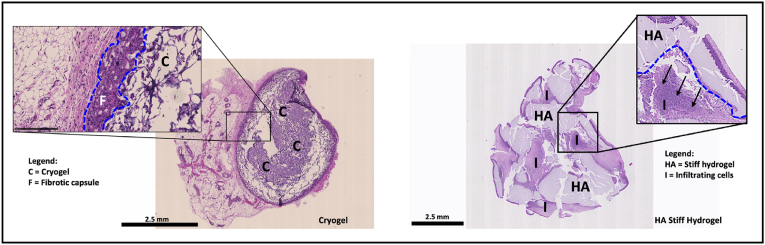
Hematoxylin and eosin (H&E) staining images of the matrices injected interperitoneally (IP) and recovered 3 days after the injection (Cryogel on the left and HA stiff hydrogel on the right). A section of the entire matrix and an inset are shown to qualitatively appreciate fibrotic capsule formation and cell infiltration. Scale bar = 2.5 mm. Images are representative of 2 animals per condition.

As shown in Fig. 6, H&E images of the matrices recovered after IP injection show the presence of resident cells migrating toward both biomaterials, likely favored by the RGD adhesive peptide functionalization. The observed cell migration is indeed crucial for gel degradation as well as for the release of nanocarriers, as previously demonstrated in-vitro. However, the initial formation of a fibrous capsule around the cryogel highlighted a foreign body response which can easily interfere with the immune response targeted by the specific immunotherapy formulation. On the contrary, the HA stiff hydrogel did not trigger any extended and acute inflammation in the injected area.

#### 2.2.2 In-vitro and in-vivo degradation

Hyaluronic acid (HA) is a biocompatible polymer and can be easily degraded in-vivo due to the presence of hyaluronidase and matrix metalloproteinases in the extracellular matrix. Heparin, another biocompatible polysaccharide, can be degraded in-vivo by heparinase enzyme, which specifically target heparan sulfate. In contrast, PEG, a widely used biopolymer, can be primarily degraded via hydrolysis, leading to the breakdown of the polymer into smaller non-toxic fragments [34,35].

A suitable degradability is needed to prevent long-term retention in the body and also to control the release of drugs and nanocarriers, as already mentioned. Preliminary in-vitro degradation studies were conducted under physiological conditions (PBS, pH = 7.4, 37 °C) by adding the hyaluronidase enzyme at a concentration of 50 U/mL to mimic the in-vivo environment. Although in-vitro tests cannot fully replicate the in-vivo behaviour, they provide valuable indications of the degradation behaviour.

**Fig. 7:**
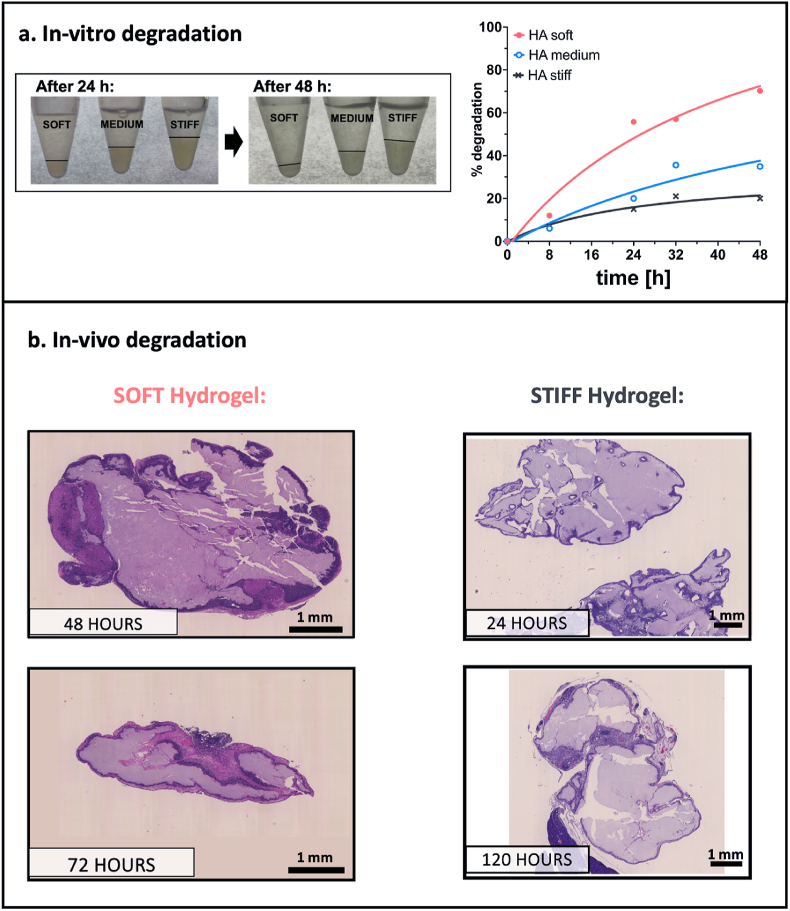
**a.** On the left residual gels are shown after 24 and 48 h of degradation in PBS with 50 U/mL hyaluronidase enzyme. In the graph (right) is reported the relative percentage of degradation of the soft (pink), medium (blue) and stiff (black) hydrogels, by weighing the dried mass of the residual hydrogel at different timepoints (0, 8, 24, 32 and 48 hours); **b**. H&E images of hydrogel residues recovered in-vivo 48 and 72 hours (left, soft gel) or 24 and 120 hours (right, stiff gel) after IP injection. Scale bar = 1 mm. Images are representative of 3 animals per condition. (For interpretation of the references to colour in this figure legend, the reader is referred to the Web version of this article.)

The hydrogels gradually degraded at different rates depending on their stiffness, which is determined by HA crosslinking and concentration. Polymer mass loss over time was quantified by collecting equal volumes of supernatant at multiple time points (0, 8, 16, 24, 32, 48 hours) and measuring the residual mass after lyophilization. As shown in Fig. 7a the tunable degradation rates ranged from approximately 20 % for the stiff formulation to 70 % for the soft formulation within 48 h.

Starting from preliminary in-vitro data, we investigated the behaviour of the matrices in-vivo by injecting two different types of gels into mice and examining the residual gel after 3 or 5 days. To better simulate the possible application of the niche, we injected 200 μL of hydrogel interperitoneally using a 26G needle. Matrices were retrieved after 24 h or 48 h and then either after 3 days for the soft gels or 5 days for the stiffer gels, to better appreciate differences in their degradability. The successful recovery of the gels indicates that they remain solid at the injection site and maintain a stable aggregated state within the body, without dispersing immediately after injection. These results, reported in Fig. 7b, demonstrate that the degradation rate can be fine-tuned by adjusting both the crosslinking degree and the HA concentration. We also evaluated, as a proof of concept, the in-vivo response of the injected niche, retrieving the soft matrix 24 h after the IP injection and performing multiplex flow cytometry to immunophenotype the main recruited innate immune cells. Among the observed cell populations, we were able to discriminate primarily dendritic cells (CD11c+), macrophages (CD11b+ and F4/80+) probably involved in the degradation of the matrix, granulocytes (CD11b+ and LY6G+), and a limited number of monocytes (CD11b+ and LY6C+) (Fig. S4).

These preliminary results highlight the ability of the material in recruiting endogenous cells required for immune system priming. Moreover, this analysis highlights the role of this retrievable niche as a valuable tool to determine the in-situ temporal evolution of the in vivo response.

## 3 Conclusion

In this work we developed an injectable, degradable soft gel (based on HA and PEG) designed to remain localized at the injection site in-vivo. This HA-based hydrogel niche showed the capability to embed and deliver multi-therapeutic components such as cells, nanocarriers and cytokines. In detail, we demonstrated that this gel can sustain the viability of primary splenocytes and create a depot for the controlled and prolonged release of biomolecules and nanocarriers through degradation or diffusion mechanisms. Our results indicate that by varying the crosslinker degree and polymer concentration, we can modulate the degradation rate of the gels from 20 % to 70 % over 48 h, adjust the mesh size from 26 nm to 67 nm, control the release of nanoparticles from 30 % to over 60 % in 120 h and regulate cytokine release by incorporating heparin, which naturally binds a variety of cytokines and chemokines.

Moreover, the currently presented HA hydrogels showed in-vivo biodegradability in addition to the absence of a severe inflammatory reaction, thereby allowing the use of this system for a targeted immune cell recruitment.

This system could be improved by using smart nanocarriers to protect drugs from enzymatic degradation and enable specific targeting, enhancing the therapeutic efficacy.

## 4 Experimental section

### 4.1 Synthesis of the injectable hydrogel

Heprasil solution (340 kDa, Advanced Biomatrix, 10 mg/mL) and Hep-SH solution (27 kDa, Creative PEGWorks, 40 mg/mL) were mixed in the proper ratio with 4arm-PEG-Ac (20 kDa, Creative PEGWorks) pre-functionalized with GRGDSPC peptide (CRIBI Biotechnology Center, University of Padova) as reported in Table S1. In detail, Heprasil solution were prepared by dissolving the lyophilized solid with degassed water. After the reconstitution, the solution was rapidly vortexed and placed horizontally on a rocker for at least 40 minutes, till the components become a clear homogeneous and slightly viscous solution. RGD peptide (21.5 mg/mL) or RDG peptide (21.5 mg/mL) was dissolved in water and pre-mixed with 4arm-PEG-Ac (200 mg/mL) dissolved in TEA buffer (0.3M, pH = 8) in order to occupy a mean of 2.5 out of 4 arms. To achieve the complete conjugation, the mix was left for 4 h at 37 °C. Then, we prepared the final hydrogel containing adjuvants in nanocarriers, cytokines, and cells, if required, by adding in order: therapeutic components, Heprasil, Heparin and as last, the PEG-RGD or PEG-RDG crosslinker. We mixed rapidly by pipetting. Then, we left the solution at 37 °C for a time necessary to reach the gelling point that we derived from the mechanical characterization.

### 4.2 Synthesis of the cryogel

Cryogels were synthetized using 4arm-PEG-Ac (20 kDa, Creative PEGWorks) pre-functionalized with GRGDSPC peptide (CRIBI Biotechnology Center, University of Padova). In detail, RGD peptide (25.9 mg/mL) was dissolved in water and mixed with 4arm-PEG-Ac (250 mg/mL) dissolved in TEA buffer (0.3 M, pH = 8) in order to achieve a molar ratio between cysteine and acrylate of 1:4. To obtain the complete conjugation, the mix was left for 4 h at 37 °C. Then, the prepolymer solutions and the precursors were precooled to 4 °C to decrease the rate of polymerization before freezing.

The polymerization occurs after the addition of 10 % w/V of ammonium persulfate (APS) and tetramethylethylendiamine (TEMED). Specifically, to prepare 500 μL of cryogel is needed: 150 μL of PEG, 32.5 μL of RGD, 306.8 μL of MilliQ, 10 μL of APS and 0.65 μL of TEMED. Once the initiator was added to prepolymer solution, the solution was quickly poured into a precooled (−20 °C) polyethylene (PE) mold and left at least 15 h at −20 °C. To obtain the cryogel, after the polymerization, the gel was thawed to remove the ice crystal and washed in milliQ water.

*Ellman's test:* To prove the RGD coupling with 4arm-PEG-Ac, the Ellman's test was performed to detect the free thiols of cysteine. Specifically, both the RGD solution and the final premix described in the section *Synthesis of the Injectable Hydrogel* were analyzed at an equivalent concentration of RGD. To perform the test, a phosphate rection buffer was prepare at a concentration of 0.1M and pH 8, supplemented 1 mM of EDTA. Ellman's reagent was then dissolved in this buffer at a concentration of 4 mg/mL. To detect the free thiols in the solution, the sample, rection buffer and Ellman's reagent were mixed in the appropriate ratio following the protocol of Thermoscientific (Ellman's reagent, Thermoscientific, catalog number 22582). The samples were incubated at room temperature for 15 min, after which the absorbance spectrum was recorded using UV-spectrophotometer (Jasco V-650 spectrophotometer). Given that Ellman's reagent is expected to emit at 412 nm, the spectra were collected over the range of 700 nm–250 nm.

### 4.3 Rheological measurement and mesh size

Rheological characterizations of hydrogels were performed with a stress-controlled rheometer (Kinexus Lab+, Netzsch, Germany). All measurements were done using a 40 mm cone-plate geometry at 37 °C with a gap of 0.5 mm. To determine the gelling kinetics of the different formulations we used a 45-min oscillatory time sweep test with a constant stress applied of 1 Pa. We monitored the evolution of the shear moduli, storage modulus (G′) and loss modulus (G″), throughout time. To determine the linear viscoelastic region (LVR) an oscillatory strain sweep was conducted in which the strain was varied in the range of 0.01%–100 % with a frequency of 1 Hz. Finally, we obtained the shear moduli using an oscillatory frequency sweep analysis in which we varied the frequency in the range of 10Hz–0.1Hz with a strain of 1 %, value found inside the LVR.

Rheological measurements of the G’ modulus can be exploited to obtain the average mesh size of hydrogels. This average mesh size (ξ, nm), representing the distance (Å) between crosslinking points, can be determined using the rubber elastic theory (RET) through the following equation:

(1) ξ=(G′NART)−13

where *G′* is the storage modulus, *N*_*A*_ is the Avogadro number (6.022 × 10^23^), *R* is the gas constant (8.314 J/K mol) and *T* is the temperature expressed in Kelvin (310 K).

*Scanning Electron Microscopy:* Scanning electron microscopy (SEM) analysis was performed on soft, medium and stiff hydrogels that have been previously incubated in PBS 1x at 37 °C for 24 h. The samples were then frozen at −80 °C and lyophilized. Prior to analysis, the samples were coated with a thin sputtered gold layer (20 nm) using Quorum Q150R E sputter coater, to charge surface and improve image quality. The coated samples were then analyzed using Joel 6490 scanning electron microscope with tungsten filament and the images were acquired at 20 keV, allowing for detailed observation of the hydrogel structure at high magnification.

### 4.4 Nanoparticles and cytokines release

Nanoparticles and cytokines release was evaluated monitoring the component released in the surnatant from the hydrogel. To determine the different rate of release due to hydrogel stiffnesses, we encapsulated, during the synthesis, fluorescent polystyrene nanoparticles with a diameter of 100 nm (Fluoresbrite Plain YG 0.1 mm Microsphere, Polysciences, Inc.). We prepared 300 μL of hydrogel in a 1.5 mL Eppendorf and we added 500 μL of buffer saline at pH = 7.4, PBS. At different timepoints (0, 8, 24, 32, 48, 120 hours) we collected 50 μL of surnatant for each hydrogel formulation (soft, medium, and stiff) and we analyzed the fluorescence intensity. For each timepoints, we replaced the collected surnatant with fresh PBS. Thanks to the calibration curve, we derived the percentage of nanoparticles released. To evaluate the efficiency of heparin binding sites we compared a formulation containing heparin with one without and we used IFN-γ as a referred cytokine. As already described above, at different timepoints (0, 1, 8, 18, 24, 48 hours) we collected 50 μL of surnatant that we replaced with fresh PBS 1x, and we revealed the amount of IFN-γ released using a specific kit ELISA.

### 4.5 Cell embedding and viability test

Immortalized human mammary epithelial cells (MCF10A cells) were cultured in DMEM:F12 supplemented with 5 % horse serum, 2 mM glutamine, EGF 20 ng/mL, insulin 10 μg/mL, cholera toxin 100 ng/mL, hydrocortisone 500 ng/mL and antibiotics. Splenocytes were obtained from murine spleen and cultured in RPMI 1640 with 10 % FBS, 2 mM glutamine, 50 μM b-mercaptoethanol and antibiotics. Cell viability was evaluated using a live/dead assay (Live/Dead Viability/Cytotoxicity Kit, for mammalian cells, Invitrogen) with calcein-AM, to detect live cells, and ethidium homodimer-1, to detect dead cells. MCF10A cells were mix at a density of 200k cell/mL inside the hydrogels and monitored for 14 days. The culture media was changed three times per week. Splenocytes were cultured in suspension, referred as standard conditions, or inside the hydrogel for 48 h at a density of 1mln cells/mL. At these timepoints cells were stained by replacing culture media with PBS 1x contained 2 μM of calcein-AM and 2 μM of ethidium homodimer-1. After 1 h of incubation at 37 °C and 5 % CO_2_ in the dark, samples were imaged with confocal microscope. At least four different fields of a 64 mm of Z-stack were acquired and a minimum of 200 cells per sample, per replicate were analyzed using Fiji.

### 4.6 In-vitro and in-vivo degradation

In-vitro degradation was investigated in physiological conditions at 37 °C in PBS at pH of 7.4 with the addition of 50 U/mL of hyaluronidase enzyme. Hydrogels of 300 μL were gelled inside a 5 mL Eppendorf, previously weighted. Then, 700 μL of the degradation buffer were added, and the gels were put in warm room under a gentle shake. At different timepoints (0, 8, 16, 24, 32, 48 hours), the surnatant was withdrawn and transferred in another 5 mL Eppendorf. Then, all the samples were lyophilized and weighted to monitor the polymer mass loss.

In vivo experiments were approved by the Italian Ministry of Health and the Committee for Research on Laboratory Animals of the University of Padova. 200 μL of soft or stiff hydrogel were injected IP in 8–10 weeks old C57BL/6 mice. Hydrogels were collected for histological examination 24h, 3 days or 5 days after the intraperitoneal injection. Collected samples were fixed in 4 % paraformaldehyde overnight and embedded in paraffin. 4 μm sections were imaged after H&E staining.

### 4.7 Multiplex flow cytometry

Immunophenotyping of endogenous recruited cells has been performed with a multiplex flow cytometry (BD FACS Celesta). The analysis has been performed on soft gels explanted 24 h after the IP injection in 8–10 weeks old C57BL/6 mice. The retrieved gels were incubated with an enzymatic solution of collagenase I (final concentration 900 U/mL) and hyaluronase (final concentration 1000 U/mL) at 37 °C on a rocker until complete digestion.

Cells were stained with a cocktail of antibodies, namely CD11c (BD, cat.n 572682), Ly6G (BD, cat.n 740953), Ly6C (BD, cat.n 561085), F4/80 (BD, cat.n 746070), CD11b (BD, cat.n 552850), CD45 (cat.n 557659), FVS700 (BD, cat.n 564997). Immune cells were initially gated on viability signal (FVS700) positivity and CD45 expression. Viable and CD45^+^ cells were then sorted to distinguish CD11c + dendritic cells from CD11b+ and F4/80+ macrophages. Monocytes and granulocytes were identified among CD11c-/CD11b + cells by Ly6C and Ly6G expression, respectively.

### 4.8 Statistical analysis

Statistically significative differences were evaluated using an unpaired *t*-test with Welch's correction (GraphPad Prism 9) and considering a confidence interval of 95 %. Confidence intervals and *p*-values are indicated in each figure legend.

## CRediT authorship contribution statement

**Veronica Torresan:** Writing – review & editing, Writing – original draft, Methodology, Investigation, Formal analysis, Data curation, Conceptualization. **Alessandro Gandin:** Validation, Investigation, Funding acquisition, Conceptualization. **Paolo Contessotto:** Writing – review & editing, Methodology, Investigation, Conceptualization. **Francesca Zanconato:** Writing – review & editing, Writing – original draft, Supervision, Resources, Methodology, Investigation, Conceptualization. **Giovanna Brusatin:** Writing – review & editing, Writing – original draft, Supervision, Resources, Funding acquisition, Data curation, Conceptualization.

## Declaration of competing interest

The authors declare that they have no known competing financial interests or personal relationships that could have appeared to influence the work reported in this paper.

## Data Availability

Data will be made available on request.
