# Supplementary material for: Injectable hyaluronic acid-based hydrogel niches to create localized and time-controlled therapy delivery
Source: Mater Today Bio. 2025 Jan 24;31:101510. doi: 10.1016/j.mtbio.2025.101510 (PMC11810838; doi:10.1016/j.mtbio.2025.101510)
Supplement: Multimedia component 1 [file mmc1.docx]

**Supporting Information**

**Injectable Hyaluronic Acid-based Hydrogel niches to create localized and time-controlled therapy delivery**

Torresan Veronica, Gandin Alessandro, Contessotto Paolo, Zanconato Francesca and Brusatin Giovanna*

***Hydrogel Compositions***

**Table S1:** Composition of materials for preparing 1 mL of final hydrogel.

| **Reagent** | **Volume (μL)**  **SOFT** | **Volume (μL)**  **MEDIUM** | **Volume (μL)**  **STIFF** |
| --- | --- | --- | --- |
| **Thiolated Hyaluronic Acid**  **[10 mg/mL]** | 400 | 650 | 650 |
| **Thiolated Heparin**  **[18 mg/mL]** | 5.5 | 2.2 | 2.2 |
| **Premix^1^** | 14.7 | 36.8 | 110.4 |
| **PBS 1x** | 329.2 | - | - |
| **Cells or PBS 1x** | 250^2^ | 311 | 237.8 |

^1^Premix: to prepare 300 μL, 162.8 μL of 4arm-PEG-Ac [200 mg/mL] were mixed with 137.2 μL of RGD peptide [21.5 mg/mL].

^2^Volume added after 30 min from the beginning of the gelation process.

***Ellman’s test***

**
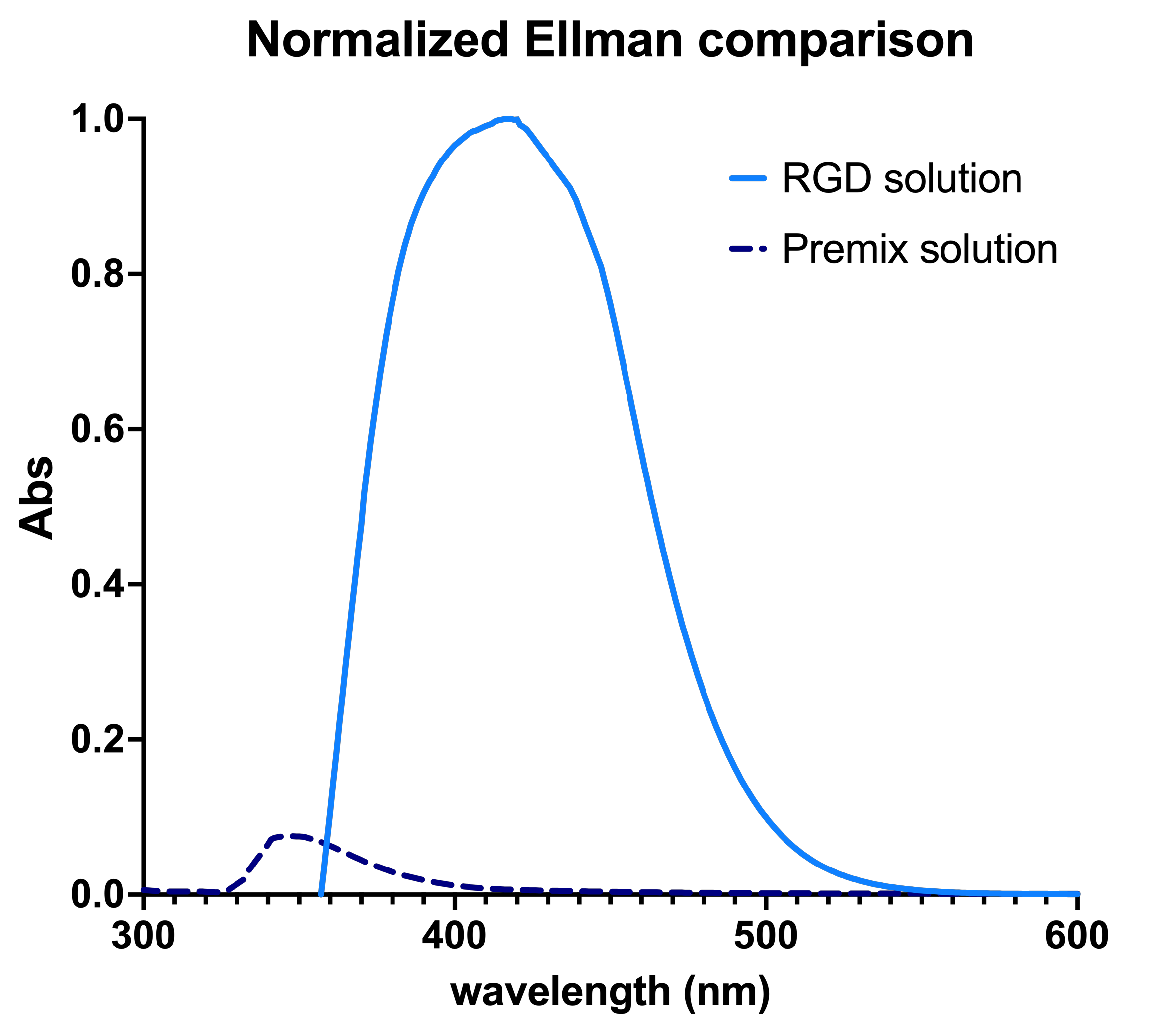
**

**Figure S1:** In the graph is reported the absorbance obtained from Ellman’s test performed on RGD solution (blue line) and premix solution (dotted line).

***SEM Analysis***

**
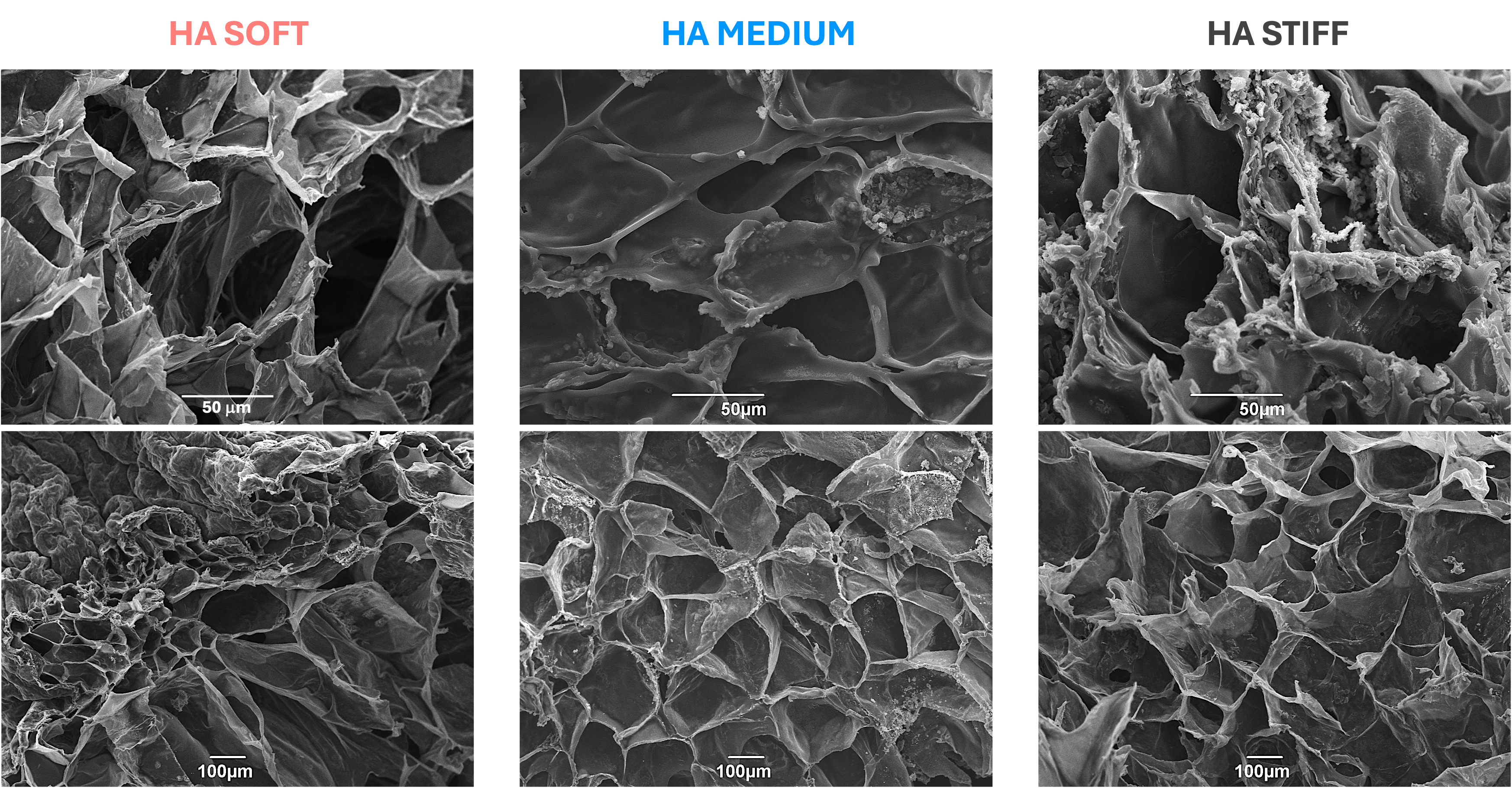
**

**Figure S2:** SEM micrographs of soft (left), medium (middle), and stiff HA (right) hydrogel. Scale bar = 50 and 100 μm.

***Nanoparticles and Cytokines Release***

**Table S2:** Means and standard deviation of nanoparticles release.

| ***Material*** | **0h** | **8h** | **24h** | **32h** | **48h** | **120h** |
| --- | --- | --- | --- | --- | --- | --- |
| **HA SOFT** | 7.66 $\pm$ 0.02 | 16.06 $\pm$ 0.82 | 37.39 $\pm$ 0.12 | 50.95 $\pm$ 0.15 | 69.67 $\pm$ 0.14 | 79.55 $\pm$0.22 |
| **HA MEDIUM** | 0.14 $\pm$ 0.00* | 3.28 $\pm$ 0.17 | 28.95 $\pm$ 0.09 | 37.51 $\pm$ 0.13 | 58.33 $\pm$ 0.13 | 70.55 $\pm$ 0.14 |
| **HA STIFF** | 0.14 $\pm$ 0.00* | 0.77 $\pm$ 0.04 | 10.54 $\pm$ 0.04 | 15.90 $\pm$ 0.04 | 24.07$\pm$ 0.05 | 28.49 $\pm$ 0.05 |

**^*^**The standard deviation is zero because no signal was detected, and the mean value corresponds to the instrument's fixed noise signal.

**Table S3:** Means and standard deviation of cytokines release.

| ***Material*** | **0h** | **2h** | **8h** | **18h** | **24h** | **48h** |
| --- | --- | --- | --- | --- | --- | --- |
| **HA SOFT** | 30.69 $\pm$ 2.99 | 23.03 $\pm$ 10.85 | 56.46 $\pm$ 1.20 | 96.15 $\pm$ 3.40 | 87.77 $\pm$ 1.95 | 72.06 $\pm$ 4.15 |
| **HA SOFT + HEPARIN** | 3.30 $\pm$ 0.19 | 3.87 $\pm$ 0.22 | 19.93 $\pm$ 0.26 | 25.38 $\pm$ 1.71 | 28.43 $\pm$ 0.05 | 27.49 $\pm$ 3.45 |

***MCF10A cell lines embedded in Soft Hydrogel***

**
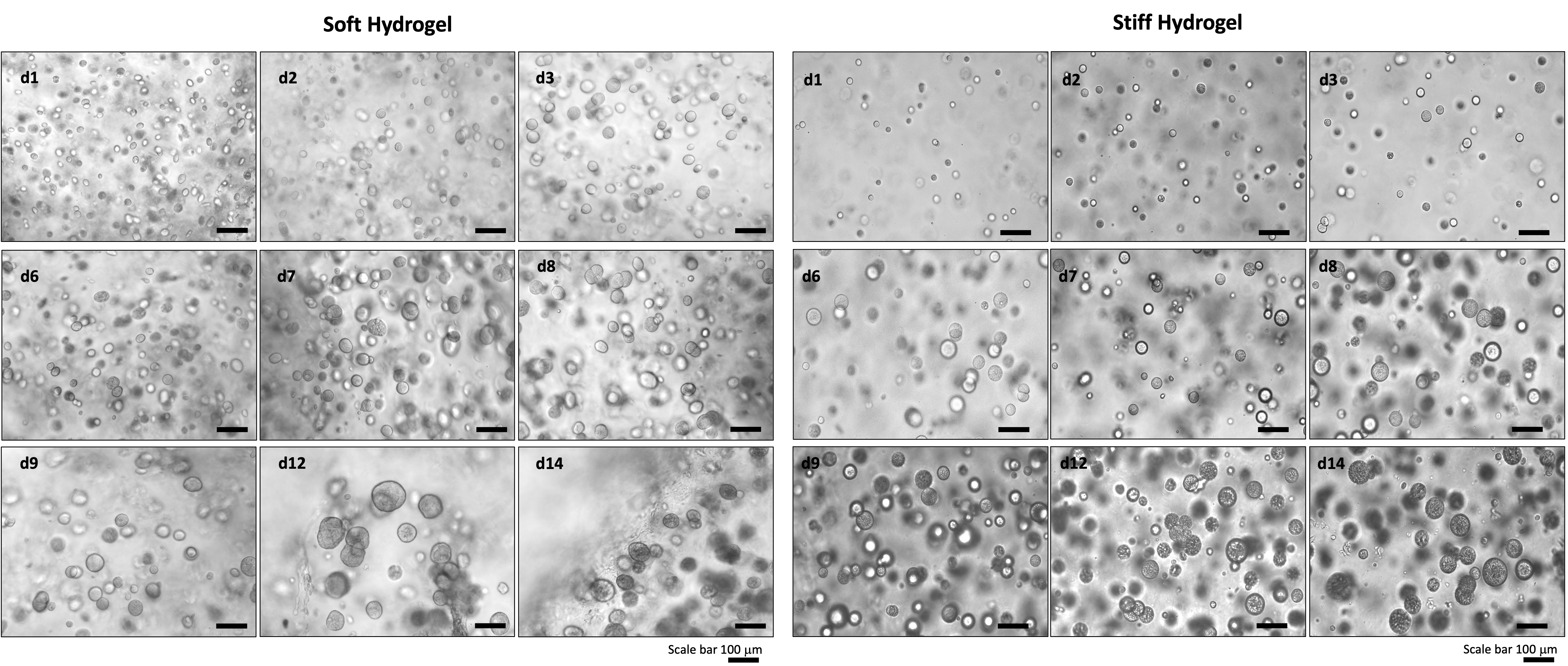
**

**Figure S3:** On the left images show MCF10A single cells embedding in Soft HA-RGD matrix for 14 days. Formation of spheroids was detected from day 2. On the right, images show MCF10A single cells embedding in stiff HA-RGD matrix for 14 days. Formation of spheroids was detected from day 3.

***Multiplex flow cytometry***

**
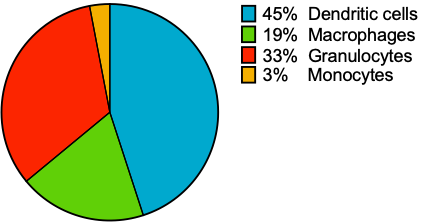
**

**Figure S4:** Cell types detected by analyzing the cellular infiltrate present into the HA soft gel through multiplex flow cytometry.

***Movie S1***

Provided as separate supplementary file.
